# An application of self-assessment of students in mathematics with intelligent decision systems: questionnaire, design and implementation at digital education

**DOI:** 10.1007/s10639-023-11761-1

**Published:** 2023-04-28

**Authors:** Vasiliki Matzavela, Efthimios Alepis

**Affiliations:** Department of Informatics, University of Piraeus, Piraeus, Greece

**Keywords:** m-learning environment, Intelligent decision systems, Predictive model, Self-assessment

## Abstract

During the last decade an eruptive increase in the demand for intelligent m-learning environments has been observed since instructors in the online academic procedures need to ensure reliability. The research for decision systems seemed inevitable for flexible and effective learning in all levels of education. The prediction of the performance of students during their final exams is considered as a difficult task. In this paper, an application is presented, contributing to an accurate prediction which would assist educators and learning experts in the extraction of useful knowledge for designing learning interventions with enhanced outcomes.

## Introduction

Τhe Artificial Intelligence in Education (AIED) has been focusing on the creating systems that are as effective, adaptive in digital education as traditional education (VanLehn, 2011) (Kochmar et al., 2021). Over 25 years, have been posted many significant papers towards that goal. Further, the ethical implications of AIED referenced in the paper of Schiff, (2021).

The Next Generation Science Standards (NGSS) have highlighted the importance of more general learning skills and competencies such as metacognition, critical thinking, and collaboration. The field of AIED correlation follow to these changes. (Roll & Wylie, 2016).

Society and technology continue to evolve given the ever-increasing access to data and information with impact across industries as well as in education. New approaches to teaching and learning prepare the graduates for demanding jobs. One of the aims of instructors is to exhibit the potential of learning through technology. The way of achieving the aforementioned is by integrating the fundamental structure of current educational systems with new technologies which require a pedagogical shift to the digital world. New education models and new content creation methods can lead to enhanced student support (Matzavela & Alepis, 2021a, 2021b).

Nowadays more emphasis has been paid to modern instructional technologies, such as online learning, blended learning and artificial intelligence, which have become increasingly important for educational projects.

The challenges posed by the pandemic of 2020 are reinforced with the growing digitization, personalization, and internationalization of education. The design of emerging virtual educational worlds opened up the opportunity for a learning experience well beyond the traditional classroom.

Machine learning is becoming more widespread and has been used for predicting students' grades, modeling student behavior, and improving curriculum design in all levels of education.(Virvou et al., 2020) Currently, the crisis caused by the Covid-19 pandemic has challenged educators from all over the world, in all areas of knowledge and educational levels, to a rapid transition in their approach to learning and teaching, leading to forced virtualization of education.(Albó et al., 2021) In this context, evaluations of the decisions, class interactions, and technological resources are needed, together with their impact on students’ competencies, such as the ability to adapt to new situations, oral and written communication, autonomy, teamwork, creativity, critical thinking, etc.(Pelánek, 2021).

The predicting of educational success is one task of the intelligent analysis of educational data. This paper is considered and focused: on improving the quality of the university students’ academic performance prediction model and implementing the developed model into the real university educational process. The system predicting academic performance is based on a merge sort algorithm. The results of the study, it was revealed make it possible to improve the quality of predicting the students’ academic performance and confirm the fact that monitoring the student’s academic performance dynamically is more helpful in making managerial decisions in the educational process. The models for predicting the student’s academic performance studied in this work can be used in educational institutions of higher education for the timely identification of at-risk students, providing feedback to students and teachers regarding the academic success of students, and managing the educational process.

Predicting student performance can help students and their teachers track their performance. Currently, an educational model is created which aims to reduce the dropout of students. Identifying underperformers at the beginning of the semester/year and increasing the attention allotted for them will aid the educational process as well as improve students’ grades. This process enables the proposed algorithm to solve discrete optimization problems without altering or hybridizing the original algorithmic framework.

During the pandemic, there was an explosive demand for online courses. Instructors have adapted the courses to the requirements and needs of each student (Frost & McCalla, 2021). Hence, the academic performance of the students, as well as the results of the final exams had to be digitally adapted and to be completed using m-learning environments. Subsequently, there was a excessive increase in the average of the students' grades in all courses. All educational institutions tried to find ways to solve the problem which was presented, with digital tools and methods aiming at sound examinations in a digital class (Baker & Hawn, 2021).

Intelligent decision systems have been employed in a plethora of sectors in our community, and blended learning has taken full advantage of predictive models in digital classes. In this approach, an algorithm was created with an intelligent decision system and a predictive model for self-assessment of students before their academic performance, focusing on their best possible preparation for every course. Self-assessment, as an important stage of the “how to learn” process, has been identified by intensive research, as a key factor of learning success, (Maldonado-Mahauad et al., 2018), (Bannert & Reimann, 2012). Assessment tasks play an important role in the selection and implementation of learning strategies, (Scouller, 1998), (Struyven et al., 2006).

In Sect. 2 the theoretical framework is presented. The architecture of the system is presented in Sect. 3, which includes the 3.1 subsection with the algorithm of the system, the 3.2 subsection which refers to the intelligent decision system, the 3.3 subsection enumerating the questionnaire, the 3.4 subsection containing the mathematical analysis, and the 3.5 subsection illustrating the user interface. Section 4 presents the conclusions of the research.

## Literature review and theoretical framework

Digital learning vs traditional learning has been promoting incorporating blended learning in schools to improve students' knowledge needs. (Boelens et al., 2018), (Harrison and West, 2014). Universities are increasing the use of blended learning because this type of learning offers flexible and effective learning.

The paper (Matzavela et al., 2017), implemented blended learning through video conferences to improve teaching–learning conditions. The purpose of the paper, (Cavanaugh et al., 2016) is to propose the use of a Learning Management System such as Sakai and Moodle to perform online evaluations at any time and place. The organization of school activities through blended learning can improve in traditional classes. (Blaine, 2019), (Prasad et al., 2018). This hybrid model of learning allows the planning of various tasks inside and outside the classroom through technology focusing to improve academic performance and developing students' skills, (Niekerk and Webb, 2016), (Yamagata-Lynch, 2014).

A lot of researchers specialize and focus in such diverse areas as artificial intelligence, fuzzy techniques, genetic algorithms, cognitive science, mathematical modeling, neural systems computer-supported cooperative work, geographic information systems, user interface management systems, informatics, knowledge representation, and applications of intelligent systems, presenting methods of modeling systems, which develop and evolve the educational process according to the student's needs (Matzavela & Alepis, 2017)(Alepis et al., 2021) (Alepis et al., 2017).

Sønderlund et al. presented a literature review specifically aimed at studying the effectiveness of learning analytics interventions based on predictive models. Of 689 papers, merely 11 studies reported an evaluation of the effectiveness of such interventions, by emphasizing the need for a solid knowledge base on the feasibility, effectiveness, and generalizability of the learning analytics interventions. Gasevic et al. present that “learning analytics is about learning.”, and recommend learning analytics and educational institutes change direction from the performance-based evaluation of learning analytics. Tempelaar et al. presented that prediction accuracy increases over time and that performance data are especially important. The number of clicks in the week before the course offered extra information and have the highest predictive power. Subsequently, the prediction of student performance gradually improved. The researchers, therefore, argued that the best time to predict student performance is as soon as possible after the first assessment. (Arruarte et al., 2021) Following the above, there is a gap in personalized learning through hybrid learning or blended learning. (Alepis & Virvou, 2014) (Virvou & Alepis, 2004) and walk step by step to optimization of learning systems.

In view of the above, this paper proposes an improved application for students with a self-assessment, which is based on a predictive model in order to more accurately predict the grade in the final exams. This application offers the student the opportunity for better preparation before the final exams, applying an intelligent decision tree system. It also offers more flexibility in this type of learning giving more opportunities for students to succeed.

## System architecture

This section and its subsections consist of the backbone of the paper. The three main parts of the architecture of an intelligent predictive model are: 1.The database (or Knowledge Base): In order to work, necessary data is needed. This data can come from a variety of sources including the Internet. In this work the data come from students of 6 Greek schools. 2.The model (eg, general decision framework and user criteria): All data collected in the database is managed by different models. These models can be standard or customized depending on the user's preferences. 3.The user interface: Another important element of the structure of a decision-making system is the user. The user communicates and interacts with the system and is considered part of it. End users alone are also very important parts of the architecture.

After creating the model, the next step is to evaluate it. To achieve this, we use test data to calculate the accuracy of the model. The model categorizes the test data. Then, the category formed on the basis of the test data is compared with the prediction made for the training data, which are independent of those of the test. The accuracy of the model is calculated from the percentage of test samples that were correctly categorized in relation to the model under training.

If the model is considered acceptable, then it can be used to categorize future data samples, the classification of which is unknown. Decision trees are widely used for categorizing and predicting data. A decision tree is constructed according to a set of pre-categorized data training. Each internal node identifies the control of the attributes and each branch that connects the internals to the offspring corresponds to a possible value for the attribute.

SubSect. 3.1 presents the classification algorithm with its parameters, subSect. 3.2 presents the intelligent decision system with binary trees, subSect. 3.3 presents the questionnaire, 3.4 the mathematical analysis and 3.5 the user interface design.

###  Algorithm

Τhe mathematician John von Neumann designed a sorting algorithm, nowadays called merge sort. It is amongst the most widely taught sorting algorithms because it epitomizes the important solving strategy known as divide and conquer: the input is split; each non-trivial part is recursively processed and the partial solutions are finally combined to form the complete solution. Whilst merge sort is not difficult to program, determining its efficiency, by means of a cost function, requires advanced mathematical knowledge. The algorithm was based on merge sort classification (Knuth, 2000). The merge sort function in classification algorithm is useful in online sorting, where the list to be sorted is taken item by item, rather than taken in its entirety from the beginning. In this application, we sort each new item we receive using any sorting algorithm, and then merge it into our sorted list using the merge sort function. However, this approach can prove to be accurate in time and space if the data is received in small chunks over the sorted list—a better approach in this case is to enter the data into a binary search tree at the time it is received.

The algorithm is as follows:

1) Initializing the variables:

“Level_1[20]” is the array that contains the 20 questions of the first level.

“Level_2[10]” is the array that contains the 10 questions of the second level.

“Level_3[6]” is the array that contains the 6 questions of the third level.

“Level_4[5]” is the array that contains the 5 questions of the fourth level.

“Grade” represents the sum of the points collected for each correct answer so far.

“MaxGrade” is the maximum grade that the student could have collected so far, and it is used in order to decide the level of difficulty of the next question, as well as for controlling when the algorithm will end.

“Level [4,3]” is a two-dimensional array that contains the number of questions and the correct answers of each level.

2) The order of the questions in each array (Level_1, Level_2, Level_3, Level_4) is randomized.

3) The first question of the first level is presented and, depending on the answer, “Grade”, “MaxGrade” and “Level [4,3]” are altered appropriately.

4) In this loop, the subroutine is called:

4.i) For each level, it checks the ratio of the correct answers to the total of questions, and if it is less than 0.5 then it selects the specific level.

4.ii) If the ratio of all levels is greater than or equal to 0.5 then the following level of the previous question is chosen.

4.a) It shows the question from the level that was chosen.

4.b) Depending on the answer, the variables “Grade”, “MaxGrade” and “Level [4,3]” are altered appropriately.

4.c) If “MaxGrade” is less than 100, the algorithm runs the same loop again.

5) When “MaxGrade” is equal to exactly 100, the final “Grade” is shown and the algorithm ends.

### Intelligent decision system

Decision systems require a structured approach. Such a framework includes people, technology, and the development approach. The Framework of Decision System consists of four phases:

Holsapple and Whinston (1996) classify Decision system into the following six frameworks: text-oriented Decision system, database-oriented Decision system, spreadsheet-oriented Decision system, solver-oriented Decision system, rule-oriented Decision system, and compound Decision system. A compound Decision system is the most popular classification for a Decision system; it is a hybrid system that includes two or more of the five basic structures.

**Fig. 1:**
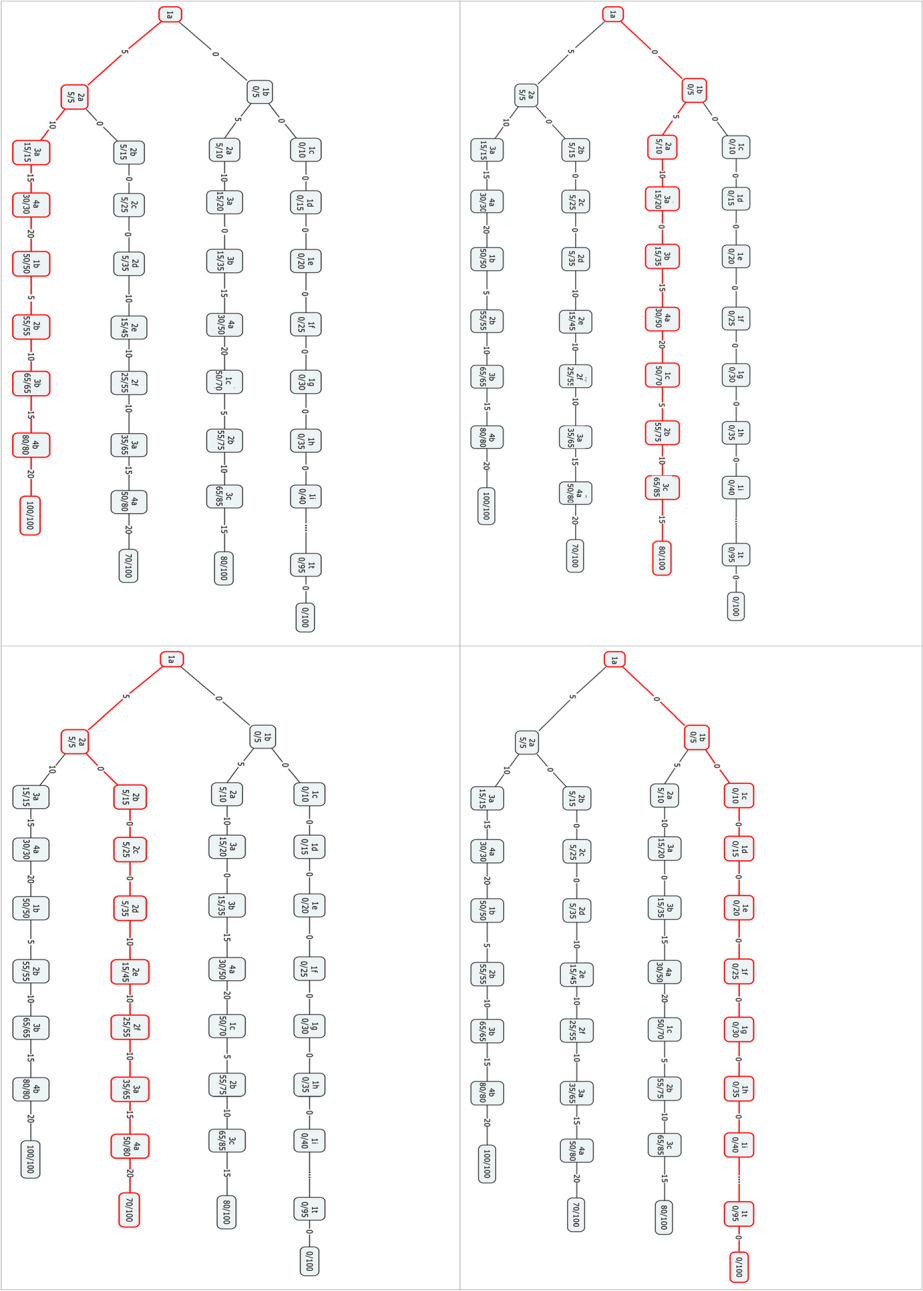
Four snapshots of the binary decision tree

Decision system components may be classified as:

The resulting binary decision tree is presented with four snapshots (Fig. 1), which illustrate students differently prepared for the Mathematics exam. Each tree has its own weights/scores per level of difficulty of the question. The questions are divided into 4 difficulty levels. In the first level of questions, there are 20 simple questions for all students. In the second level of questions, there are 10 questions of moderate difficulty. In the third level of questions, there are 6 questions that are more demanding for students. In the fourth level, there are 5 questions of the high cognitive field for the completion of the test. The points for each of the first level questions are 5 points, for the second level 10 points, for the third 15 points and for the fourth 20 points. The final score is 100/100 for the excellently prepared student. In the first picture, we see the excellent student, who correctly answered questions from all levels (8 questions in total), in random order, and he/she collected 100/100 points. In the second picture, we see a student who answers 9 questions and collected 80/100 points. In the third picture, we see a different combination of questions, and the student collected 70/100 points. The fourth picture shows a student who has not been prepared for this exam and his/her score was 0/100.

### Questionnaire

This questionnaire was based on Mathematics, under the supervision of Mathematical Scientists, and on specific chapters. However, the following questionnaire can be implemented with the same algorithm and the integrated predictive model in all courses with similar questions (Tables 1, 2, 3).

**Table 1:** The questions of all levels

	Questions		Questions
1.a	Express the commutative property of the addition	2.a	Expand the expression: (-3x-5)^2^
1.b	Complete the equality: a^m^a^n^ =	2.b	Simplify the following expression: (11x^2^y-12w^3^)(11x^2^y + 12w^3^)
1.c	If $\frac{x}{y}=2$, calculate the value of the expression:$A=\frac{{\left({x}^{2}{y}^{-2}\right)}^{2}{\left({x}^{3}y\right)}^{-1}}{{\left({x}^{2}{y}^{-1}\right)}^{-2}}$	2.c	If $x-\frac{1}{x}=-2$, calculate the expression: ${x}^{2}+\frac{1}{{x}^{2}}$
1.d	Is the following statement true or false:If α, b positive numbers, then $\sqrt{a}+\sqrt{b}=\sqrt{a+b}$	2.d	If the numbers x, y are inverse, calculate the expression: A = (x + 2y)^2^ – (2x-y)^2^ + 3x^2^ – 3y^2^
1.e	Calculate the value of the expression:$\Delta =\sqrt{21+2\sqrt{1+\sqrt{9}}}$	2.e	Write the following expression in polynomial form: A(x) = (x-1)(x + 1)(x^2^ + 1)-(x^2^-1)
1.f	Solve the equation:$\frac{x\sqrt{5}}{\sqrt{2}}=\sqrt{8}$	2.f	Factorize the expression: 4x^2^(x-2)-12x(x-2) + 9x-18
1.g	Perform the algebraic operations: (-2x^2^)^3^ + (-x^3^)^2^ + 7x^6^	2.g	Calculate the value of the expression: A = 55·87 – 55·32 – 45^2^
1.h	Expand the following expression:${\left(\sqrt{2}+\sqrt{5}\right)}^{2}$	2.h	Factorize the expression: (x-1)^2^ – 6(x-1) + 9
1.i	Calculate the value of the arithmetic expression: $\frac{1}{\left(2-\sqrt{3}\right)\left(2+\sqrt{3}\right)}$	2.i	Factorize the trinomial: x^2^ – 6x + 8
1.j	Write the following expression in polynomial form: P(x) = (x-1)^2^-(3x-2)^2^-2x(5-4x)	2.j	Simplify the expression:$\frac{{x}^{2}-4+x\left(x-2\right)}{2{\mathrm{x}}^{2}-8\mathrm{x}+8}$
1.k	Factorize the expression: (x-1)(x^2^-9)-(x + 3)(x^2^-1)	3.a	Is the following statement true or false:$\frac{x+2\mathrm{y}}{z+2\mathrm{y}}=\frac{x}{z}$
1.l	If $x=-\frac{2}{3}$, calculate the value of the expression: A = 9x^3^-3x^2^-5x-1	3.b	Find the product:$\frac{{x}^{2}-1}{{x}^{2}-3\mathrm{x}}\bullet \frac{{x}^{2}-9}{3\mathrm{x}-3}$
1.m	If $\frac{{55}^{v}{3}^{v}}{{33}^{v}}=625$, find the value of v	3.c	Perform the algebraic operations:$\frac{y}{y-1}\bullet \frac{{y}^{2}-36}{y+1}\div \frac{y+6}{y+1}$
1.n	If we double the side of a square, how many times does its area increase?	3.d	Perform the algebraic operation:$\frac{3\mathrm{x}}{x-1}-\frac{x+2}{x-1}$
1.o	Calculate the expression:$\sqrt{2}\sqrt{8}+\sqrt{36\bullet 121}$	3.e	Perform the algebraic operation:$\left(1-\frac{2\mathrm{x}}{{x}^{2}+1}\right)\left(x+\frac{x+1}{x-1}\right)$
1.p	Simplify the following fraction giving the result with a rational denominator:$\frac{6}{\sqrt{48}}$	3.e	Solve the equation: $\frac{2\mathrm{x}-1}{3}-\frac{x-2}{6}=x-2$
1.q	Prove that $\frac{3\sqrt{2}\sqrt{6}+\sqrt{6}\sqrt{8}}{\sqrt{5}\sqrt{15}}=2$	4.a	Solve the equation: 1-2x(x-1) = 1-2x
1.r	If A(x) = x^3^-3x^2^-3 × and B(x) = 2x^3^ + 3x^2^-2x + 1 find the polynomial: C(x) = A(x)-B(x)	4.b	Solve the equation: (2x-1)^2^ – x(x-1) = 2 + x^2^
1.s	Write the following expression in polynomial form: Q(x) = 1-(2x + 1)(5x-3)	4.c	Simplify the expression:$\frac{{3\mathrm{x}}^{2}-5\mathrm{xy}-{2\mathrm{y}}^{2}}{{9\mathrm{x}}^{2}-{4\mathrm{y}}^{2}}$
1.t	If P(x) = x^3^-3 × and Q(x) = x^2^-2 find the product: D(x) = P(x)·Q(x)	4.d	Solve the equation:$\frac{1}{{x}^{2}-5\mathrm{x}+6}-\frac{1}{x-3}=\frac{1}{{x}^{2}-4\mathrm{x}+4}$
		4.e	Solve the inequality:$1-\frac{2\mathrm{x}-1}{6}<\frac{x-2}{3}-\frac{x}{2}$

**Table 2:** Assessment of the application(percentage analysis)

Q1	Do you like the application?	Yes: 97.17%	No: 2.83%
Q2	Was your performance improved?	Yes: 96.61%	No: 3.39%
Q3	Was it easy to use?	Yes: 98.87%	No: 1.13%
Q4	Would you recommend it to others?	Yes: 96.04%	No: 3.96%

**Table 3:**
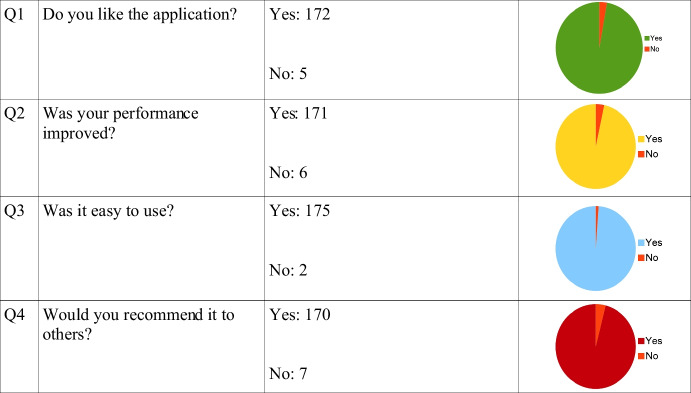
Quantitative analysis

The questionnaire consists of questions of four different levels for self-assessment in Mathematics. The whole cognitive background for the specific course is examined prior to the final exam, allowing the students to improve their performance, while the institutions can extract useful educational results. The questionnaire consists of questions in random order for each student, in order to prevent, as far as possible, cheating in the test and for the grades to remain objective. The question types are 3: multiple-choice, true/false, and open text.

### Mathematical analysis

The intelligent decision system first solves two important problems, and their solutions are defined below, in order to predict the number of different outputs provided by the system.

Problem 1: We have 4 levels of questions: level 1 contains l_1_ = 20 questions of value 1, level 2 contains l_2_ = 10 questions of value 2, level 3 contains l_3_ = 6 questions of value 3 and level 4 contains l_4_ = 5 questions of value 4. Let *S*_20_ be the number of questions sets that can be formed such that the total number of questions equals 20. If k_1_, k_2_, k_3_, k_4_ is the number of questions selected from levels 1, 2, 3, 4 respectively, then it is clear that these numbers satisfy.k_1_ + 2k_2_ + 3k_3_ + 4k_4_ = 20 (1)

Moreover, there exist $$\left(\begin{array}{c}{l}_{i}\\ {k}_{i}\end{array}\right)$$ ways to choose ki questions from level $i\in \left\{\mathrm{1,2},\mathrm{3,4}\right\}$

More formally *S*_20_ is the number of partitions of 20 into parts no greater than 4, where k_i_ counts the number of parts equal to *i* and each partition of type (k_1_, k_2_, k_3_, k_4_) has weight

$$w\left({k}_{1},{k}_{2},{k}_{3},{k}_{4}\right)=\prod_{i=1}^{4}\left(\begin{array}{c}{l}_{i}\\ {k}_{i}\end{array}\right)$$

For example, a valid partition is 1 + 1 + 1 + 3 + 3 + 3 + 4 + 4 = 20, its type is (k_1_, k_2_, k_3_, k_4_) =  (3, 0, 3, 2) and it corresponds to *w*(3, 0, 3, 2) different question sets. Each such question set contains 3 level 1, 3 level 3 and 2 level 4 questions. Note that these questions can be ordered in $\frac{\left({k}_{1}+{k}_{2}+{k}_{3}+{k}_{4}\right)!}{{k}_{1}!{k}_{2}!{k}_{3}!{k}_{4}!}$ different ways but, under the above definition, the order of the questions is irrelevant, since *S*_20_ counts question sets and not question lists. Therefore,

$${S}_{20}=\sum_{{k}_{1}+2{k}_{2}+3{k}_{3}+4{k}_{4}}\prod_{i=1}^{4}\left(\begin{array}{c}{l}_{i}\\ {k}_{i}\end{array}\right)=\left[{x}^{n}\right]\prod_{i=1}^{4}\sum_{k\ge 0}\left(\begin{array}{c}{l}_{i}\\ {k}_{i}\end{array}\right){x}^{{ik}_{i}}=\left[{x}^{n}\right]\prod_{i=1}^{4}{\left(1+{x}^{i}\right)}^{{l}_{i}}$$

Denote by *f*(*x*) the product of the last equality, i.e.,

$$f\left(x\right)={\left(1 + x\right)}^{20}{\left(1 + x2\right)}^{10}{\left(1 + {x}^{3}\right)}^{6}{\left(1 + {x}^{4}\right)}^{5}$$

It follows that

$${S}_{20}=\left[{x}^{20}\right]f\left(x\right)=2845201114$$

The above coefficient is easily computed using any symbolic computation software.

Problem 2: In the above problem, we allowed zero values for any of the k_i_’s. But what if each question requires that at least one question from every lower level is contained in the solution? Let *S*'_20_ denote the number of solutions in this case. In order to solve this problem, we define

$${f}_{i}\left(x\right)=\prod_{j=1}^{i}\left({\left(1+{x}^{j}\right)}^{{l}_{j}}-1\right),i\in \left\{\mathrm{1,2},\mathrm{3,4}\right\}$$

so that the coefficient of *x*^20^ in *f*_*i*_(*x*) equals the number of solutions containing questions of maximum level *i*. Then, we can calculate as before,

$$\left[x^{20}\right]f_1(x)\:=\:1\left[x^{20}\right]f_2(x)\:=\:109,208,162,\left[x^{20}\right]f_3(x)\:=\:1,158,309,895,\left[x^{20}\right]f_4(x)\:=\:1,061,692,900.$$

and

$$S_{20}^{\prime}\:=\:\left[x^{20}\right]\left(f_1\left(x\right)\:+\:f_2\left(x\right)\:+\:f_3\left(x\right)\:+\:f_4\left(x\right)\right)\:=\:2,329,210,958.$$

### User Interface Design and evaluation

The user interface of the application was created with the aim of being user-friendly and providing flexibility and adaptability to the needs of students. A self-assessment provides a clear picture of the student's preparation for the lesson. By using the application, the student knows in advance his performance and his rate of self-improvement. The goal is to reduce the percentage of students who drop out or repeat the course, which is time consuming and costly.

In the beginning, the ID of the certificated student is checked, and the timer of the process is initialized. The remaining time is presented on all pages of questions and is following the type of answers. The final score is disclosed on the last page, without delay. The benefits to the students appeared when the application was widely used in 6 Greek schools. Before each competition they knew their performance which was proportional to the study.

**Fig. 2:**
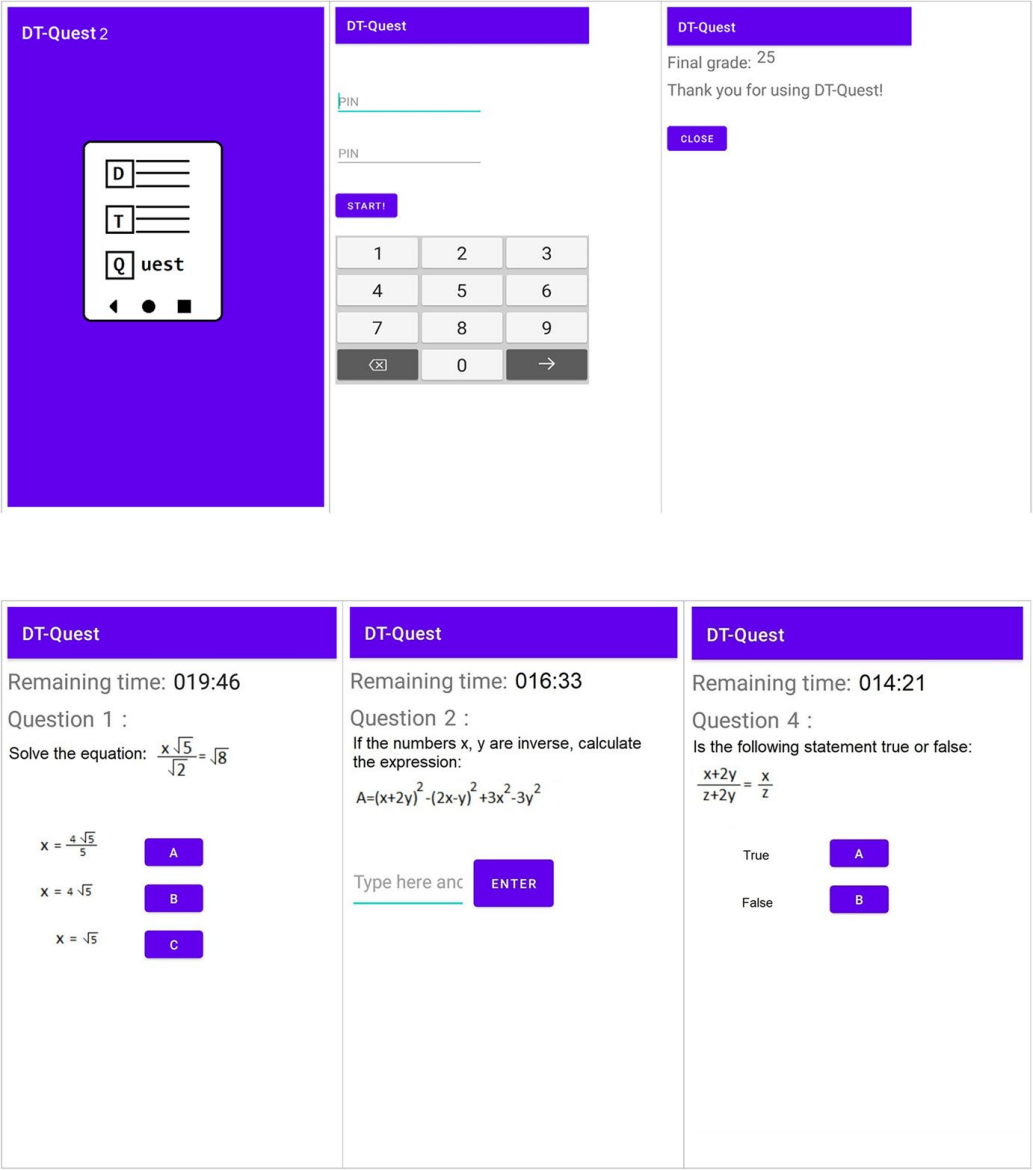
Using the application

In the first row of (Fig. 2), the initial screen of the application is displayed in the first image on the left, then the second image displays the screen where students type their PIN, which ensures their identification. The last screenshot of the first row displays the last screen that the students will encounter, where their final grade of the self-assessment is shown. In the second row, in the first picture on the left, the screenshot of question 1 is displayed, as well as the remaining time. The type of this specific question provides a multiple-choice answer. The next screenshot displays question 2 where the students can write text for the answer. The last screenshot of the second row displays question 4 with the options right/wrong.

The exercises were given to 177 Mathematics students from 6 Greek schools, with a questionnaire for evaluation. The results were encouraging when asked whether they liked the application with a percentage of yes 97.17%. The next question is whether their performance was improved with a percentage of yes 96.61%, whether it was easy to use with a percentage of yes 98.87% and whether they would recommend it to others with a percentage of yes 96.04%.

The resulting system was presented to and evaluated by students, through the completion of questionnaires.

The quantitative analysis follows:

Subsequently, by analyzing the enthusiastic results, the utilization and feasibility of the application were confirmed. Such an application can find space in learning environments for students' personalized needs. The use of analytics improves the overall learning design quality and helps educators avoid committing design errors.

## Conclusions

The main aim of the paper is the prediction of students' academic performance before/after the final exams through intelligent decision systems. Before the exams, the self-assessment provides to students’ enhancement of their performance while focusing on the difficult study points. After the exams, each institution determines the individual profile of the student and his/her knowledge needs in digital class and m-learning environments.

The specific app focused on Mathematics, while it could be useful for all lessons with the appropriate parameterization of the questions. (Virvou et al., 2013).

The structure of the system has been supported by a mathematical analysis where the number of combinations of random questions is analyzed. Due to the vast number of combinations of questions, the risk of students cheating in the exam is minimized.

According to the paper (Matzavela & Alepis, 2017), the major percentage of learners of all different age groups prefer adaptive learning in a physical class, whereas digital education influences the students' attributes and the dropout in courses. For the above reasons, digital classroom learning needs to be optimized at the classroom and examination level. The paper (Matzavela & Alepis, 2021a, 2021b) states that the decision tree method has various advantages: It is simple to understand and interpret, it is easy to display graphically, and it is capable to handle both numerical and categorical data.

The implementation of this app in digital education has been evaluated by students with excellent results. The present study offers improvements in students' self-assessment, which positively affects their performance, and reduces rejections in a course by institutions. This application can be integrated into online learning, such as mobile learning, hybrid learning or blended learning. aiming for an intelligent decision system with a predictive model that accurately predicts a student's optimal grade.

Subsequently, the DT-Quest 2 app was created according to the above benefits for students and institutions for m-learning environments. Future studies could be based on intelligent decision systems, (a path of machine learning), which contribute to all fields of education as well as of economics, business, medicine, etc. Institutions, by focusing on studies that accurately predict student grades, enhance the quality of the studies they provide, and minimize dropouts.

## Data Availability

All data generated or analyzed during this study are included in this published article (and its supplementary information files).

## References

[CR1] Albó, L., Barria-Pineda, J., Brusilovsky, P., & Hernández-Leo, D. (2021). Knowledge-Based Design Analytics for Authoring Courses with Smart Learning Content. *International Journal of Artificial Intelligence in Education*, 1–24.

[CR2] Alepis, E., Kabassi, K., & Virvou, M. (2017, November). Personalized museum exploration by mobile devices. In Interactive Mobile Communication, Technologies and Learning (pp. 353–360). Springer, Cham.

[CR3] Alepis E, Virvou M (2014). Object-oriented user interfaces for personalized mobile learning.

[CR4] Alepis, E., Maria, V., & Kontomaris, P. (2021, July). Covid-19 Mobile Tracking Application Utilizing Smart Sensors. In 2021 12th International Conference on Information, Intelligence, Systems & Applications (IISA) (pp. 1–8). IEEE.

[CR5] Arruarte J, Larrañaga M, Arruarte A, Elorriaga JA (2021). Measuring the Quality of Test-based Exercises Based on the Performance of Students. International Journal of Artificial Intelligence in Education.

[CR6] Baker, R. S., & Hawn, A. (2021). Algorithmic bias in education. *International Journal of Artificial Intelligence in Education*, 1–41.

[CR7] Bannert M, Reimann P (2012). Supporting self-regulated hypermedia learning through prompts. Instructional Science.

[CR8] Blaine AM (2019). Interaction and presence in the virtual classroom: An analysis of the perceptions of students and teachers in online and blended Advanced Placement courses. Computers & Education.

[CR9] Boelens R, Voet M, De Wever B (2018). The design of blended learning in response to student diversity in higher education: Instructors’ views and use of differentiated instruction in blended learning. Computers & Education.

[CR10] Cavanaugh, C., Hargis, J., & Mayberry, J. (2016). Participation in the virtual environment of blended college courses: An activity study of student performance. The International Review of Research in Open and Distributed Learning, 17(3).

[CR11] Frost S, McCalla G (2021). A planning algorithm to support learning in open-ended, unstructured environments. International Journal of Artificial Intelligence in Education.

[CR12] Gašević D, Dawson S, Siemens G (2015). Let’s not forget: Learning analytics are about learning. TechTrends.

[CR13] Harrison JB, West RE (2014). Sense of community in a blended technology integration course: A design-based research study. International Review of Research in Open and Distributed Learning.

[CR14] Holsapple, C.W., and A. B. Whinston. (1996). Decision Support Systems: A Knowledge-Based Approach. St. Paul: West Publishing. ISBN 0-324-03578-0.

[CR15] Knuth, D. (2000) Selected Papers on the Analysis of Algorithms, chapter Big Omicron and Big Omega and Big Theta, pages 35–41. CSLI Publications.

[CR16] Kochmar, E., Vu, D. D., Belfer, R., Gupta, V., Serban, I. V., & Pineau, J. (2021). Automated Data-Driven Generation of Personalized Pedagogical Interventions in Intelligent Tutoring Systems. *International Journal of Artificial Intelligence in Education*, 1–27.

[CR17] Larrabee Sønderlund A, Hughes E, Smith J (2019). The efficacy of learning analytics interventions in higher education: A systematic review. British Journal of Educational Technology.

[CR18] Maldonado-Mahauad, J., Pérez-Sanagustín, M., Moreno-Marcos, P. M., Alario-Hoyos, C., Muñoz-Merino, P. J., & Delgado-Kloos, C. (2018, September). Predicting learners’ success in a self-paced MOOC through sequence patterns of self-regulated learning. In European conference on technology enhanced learning (pp. 355–369). Springer, Cham.

[CR19] Matzavela, V., & Alepis, E. (2017). A survey for the evolution of adaptive learning in mobile and electronic devices. In 2017 8th International Conference on Information, Intelligence, Systems & Applications (IISA) (pp. 1–5). IEEE.

[CR20] Matzavela, V., Chrysafiadi, K., & Alepis, E. (2017, April). Questionnaires and artificial neural networks: a literature review on modern techniques in education. In *2017 IEEE Global Engineering Education Conference (EDUCON)* (pp. 1700–1704). IEEE.

[CR21] Matzavela, V., & Alepis, E. (2021a, November). Decision tree learning through a predictive model for student academic performance in intelligent m-learning environments. Computers and Education: Artificial Intelligence, 2, 100035.

[CR22] Matzavela, V., & Alepis, E. (2021b, August). M-learning in the COVID-19 era: physical vs digital class . Education and Information Technologies, 1–21. 10.1007/s10639-021-10572-6 PMC8106359 33994833

[CR23] Pelánek, R. (2021). Adaptive, Intelligent, and Personalized: Navigating the Terminological Maze Behind Educational Technology. International Journal of Artificial Intelligence in Education, 1–23.

[CR24] Prasad PWC, Maag A, Redestowicz M, Hoe LS (2018). Unfamiliar technology: Reaction of international students to blended learning. Computers & Education.

[CR25] Roll I, Wylie R (2016). Evolution and revolution in artificial intelligence in education. International Journal of Artificial Intelligence in Education.

[CR26] Schiff, D. (2021). Education for AI, not AI for Education: The Role of Education and Ethics in National AI Policy Strategies. *International Journal of Artificial Intelligence in Education*, 1–37.

[CR27] Scouller K (1998). The influence of assessment method on students' learning approaches: Multiple choice question examination versus assignment essay. Higher Education.

[CR28] Struyven K, Dochy F, Janssens S, Schelfhou W, Gielen S (2006). The overall effects of end-of-course assessment on student performance: A comparison between multiple choice testing, peer assessment, case-based assessment and portfolio assessment. Studies in Educational Evaluation.

[CR29] Tempelaar DT, Rienties B, Giesbers B (2015). In search for the most informative data for feedback generation: Learning analytics in a data-rich context. Computers in Human Behavior.

[CR30] VanLehn K (2011). The relative effectiveness of human tutoring, intelligent tutoring systems, and other tutoring systems. Educational Psychologist.

[CR31] Van Niekerk J, Webb P (2016). The effectiveness of brain-compatible blended learning material in the teaching of programming logic. Computers & Education.

[CR32] Virvou, M., & Alepis, E. (2004, October). Mobile versus desktop facilities for an e-learning system: users' perspective. In 2004 IEEE International Conference on Systems, Man and Cybernetics (IEEE Cat. No. 04CH37583) (Vol. 1, pp. 48–52). IEEE.

[CR33] Virvou, M., Alepis, E., & Mpalasis, K. (2013). Evaluation of a multimedia educational tool for geography in elementary schools. In Proceedings of International Conference on Information Communication Technologies in Education, ICICTE (pp. 364–374).

[CR34] Virvou, M., Alepis, E., Tsihrintzis, G. A., & Jain, L. C. (2020). Machine learning paradigms. In Machine Learning Paradigms (pp. 1–5). Springer, Cham.

[CR35] Wang, Q., Quek, C. L., & Hu, X. (2017). Designing and improving a blended synchronous learning environment: An educational design research. The International Review of Research in Open and Distributed Learning, 18(3).

[CR36] Yamagata-Lynch LC (2014). Blending online asynchronous and synchronous learning. International Review of Research in Open and Distributed Learning.

